# Fabrication of doxorubicin-gated mesoporous polydopamine nanoplatforms for multimode imaging-guided synergistic chemophotothermal therapy of tumors

**DOI:** 10.1080/10717544.2020.1730523

**Published:** 2020-02-24

**Authors:** Min Yang, Ningnan Zhang, Tao Zhang, Xian Yin, Jie Shen

**Affiliations:** a Department of Urology, The First People’s Hospital of Yunnan Province, Kunming University of Science and Technology, Kunming, Yunnan, P. R. China;; b School of Chemical Science and Technology, Yunnan University, Kunming, Yunnan, P. R. China

**Keywords:** Mesoporous polydopamine, nanotheranostics, ultrasound imaging, drug gatekeeper, synergistic therapy

## Abstract

A versatile theranostic agent that integrated with therapeutic and diagnostic functions is extremely essential for cancer theranostic. Herein, a multifunctional theranostic nanoplatform (PFP@MPDA-DOX) based on perfluoropentane (PFP) encapsulated mesoporous polydopamine (MPDA) is elaborately designed, followed by gating of drug doxorubicin (DOX) for preventing cargo leaking. The MPDA with pH-responsive biodegradation behavior was served as nanocarrier, which also endows the nanoplatform with a large cavity for PFP filling. The nanoparticles were then gated with DOX molecule by Michael addition and/or Schiff base reaction to shield the leaking of PFP during the blood circulation before the tumor tissue is reached. Also, such nanotheranostic exhibits high photothermal conversion efficiency of 45.6%, which can act as an intelligent nanosystem for photothermal therapy (PTT) and photoacoustic (PA) imaging. Moreover, the liquid-gas phase transition of PFP arising upon exposure to an 808 nm laser and thus produced the bubbles for ultrasound (US) imaging. The subsequent PFP@MPDA-DOX-mediated synergetic chemotherapy (contributed by the DOX gatekeeper) and PTT (contributed by the MPDA) shows excellent anticancer activity, which has been systematically evaluated both *in vitro* and *in vivo*. All these positive results certify that the facile incorporation of the antitumor drug gatekeeper and MPDA into one theranostic nanoplatform shows general potential for multimode PA/US imaging and combination chemotherapy/PTT of tumors.

## Introduction

To integrate therapeutic and diagnosis elements into single nanoplatform is a good strategy for improving therapeutic outcome in cancer theranostics. Until now, a variety of nanomaterials-based systems have been developed for improving theranostic efficacy of different diseases (Kiessling et al., 2014; Lim et al., 2015; Qi et al., 2018). For instance, 2D nanomaterials (e.g. graphene (Sun et al., 2008), transition-metal dichalcogenides (Dong et al., 2016), black phosphorus (Tao et al., 2017) and boron nanosheets (Ji et al., 2018)), magnetic nanoparticles (NPs) (Tian et al., 2017), semiconductor nanocrystals (Yu et al., 2018), mesoporous nanomaterials (Wu et al., 2018), calcium-based nanomaterials (Qi et al., 2018), and some organic nanomaterials (Xiao et al., 2018; Li et al., 2019a) have been extensively explored. Among of them, mesoporous nanomaterials with easy surface functionalization, large surface areas, well-defined morphologies, together with high payload capability and remarkable biocompatibility, endow them promising for fabrication of theranostics nanoplatform (Mekaru et al., 2015; Li et al., 2017; Xu, Han, et al., 2018). Thanks to these general prosperities, the utility of mesoporous silica (MSN) in cooperation with other cargo molecules or inorganic NPs has been widely implemented in the previous studies, and thus a variety of MSN-based nanosystems have been extensively designed for precise drug delivery and theranostics (Zheng et al., 2015; Chen et al., 2017; Lu et al., 2017). Although mesoporous NPs have attracted extensive research in nanomedicine, the research of these nanomaterials is still ahead some unpredictable problems. The main challenge is the biosafety and biodegradation of inorganic nanostructure, which substantially limiting their successful clinical transformation (Ehlerding et al., 2016; Croissant et al., 2017). Thus, a new mesoporous nanocarrier with specific biodegradability and low potential risks is highly anticipated.

Aiming at the advantages of organic-inorganic hybrid, mesoporous organosilicas (MONs) that integrated organic groups within the framework of silica have been constructed to achieve the tumor-specific biodegradability of mesoporous nanomaterials, which have been proved in recent years (Huang et al., 2017; Lu et al., 2018). More importantly, the integration of MONs with other therapeutic or diagnosis modalities have been extensively studied and shown great potential in cancer theranostics (Chen, Meng, et al., 2014; Li et al., 2018b). Inspired by this, new mesoporous nanostructures, namely mesoporous polydopamine (MPDA) NPs have been constructed. As a melanin-like polymer, PDA has been widely served as a versatile material for biomedical applications (Chen et al., 2016; Liu et al., 2016). Typically, PDA coating has been demonstrated to be an ideal strategy for improving the stability of the host materials due to the excellent biodegradation and biocompatibility of PDA. For instance, the PDA coating with pH responsiveness was used by Zeng and coworkers as a gatekeeper to cap MSNs to fabricate the drug delivery system for cancer synergistic therapy. Besides, the strong near-infrared (NIR) absorption of PDA enables it can be acted as a photothermal conversion agent for photothermal therapy (PTT) and photoacoustic (PA) imaging (Cheng et al., 2017). Based on these significant advantages, MPDA NPs with pore structures exhibits an efficient drug loading, as well as could efficiently produce mild hyperthermia for PTT (Xing et al., 2017; Wu, Duan, et al., 2019a). Despite these gratifying developments, the use of MPDA NPs as nanocarriers are still lacking for synergistic thermo-chemotherapy.

To design a mesoporous-based drug delivery system, a crucial and prominent factor is selecting an appropriate controlled gatekeeper to cap the pore entrances, thus shielding the leaking of payloads during the blood circulation before the tumor tissue is reached (Wen et al., 2017). To date, a number of capping agents, including polymers (Zhang et al., 2015), inorganic nanomaterials (Lu et al., 2017), host-guest assemblies (Lei et al., 2016), and biomacromolecules (Zhang et al., 2013) have been employed to fabricate the MSN-based controlled stimuli-responsive nanoplatform. However, the bioresponsiveness of most gatekeepers will be sacrificed after modification with the MSN. Also, some unpredictable toxicity of the gatekeeper agents will further restrict its clinical translation. Thus, it is a challenge to fabricate intelligent nanosystems based on mesoporous nanomaterial with no potential risks gatekeeper and simultaneously realizing on-demand drug release. Zeng and coworkers reported a prominent work by using the drug itself as gatekeeper to minimize the unpredictable toxicity of subsidiary sealing agents, as well as provides a general strategy for the fabrication of drug-self-gated nanoplatform (Zeng et al., 2017). Based on this, a few recent studies have proved that drug-self-gated is expected be a desirable strategy for the construction of novel mesoporous nanostructures-based nanoplatform (Wu et al., 2018; Wu, Williams, et al., 2019b). Thus, it is conceivable that design a new nanosystem based on MPDA that are capped by drug molecules is feasible.

**Figure 1.**
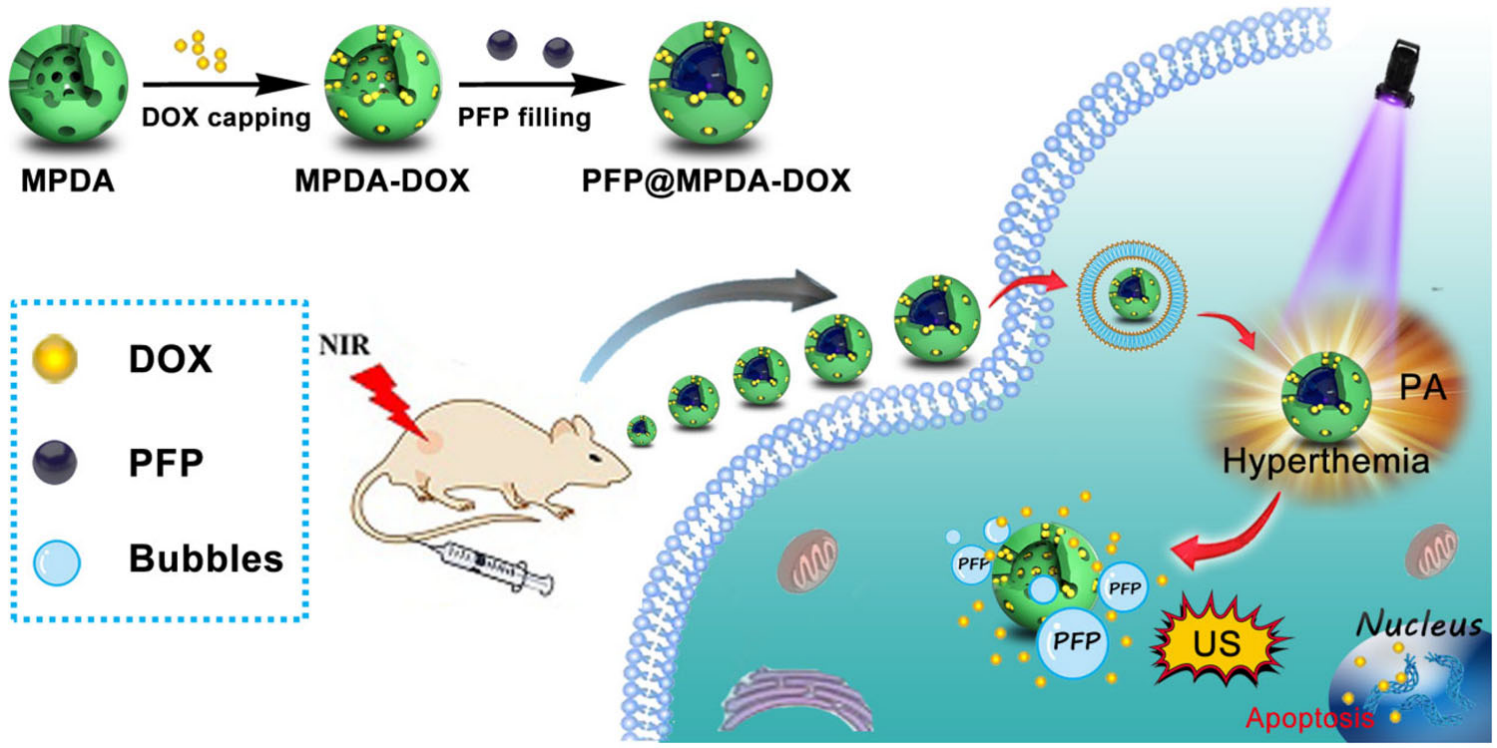
Schematic illustration of the procedure used to fabricate PFP@MPDA-DOX nanotheranostics for PA/US guided chemo-photothermal therapy of tumor.

Herein, we design a versatile nanotheranostics agent based on doxorubicin (DOX)-gated MPDA for multimode imaging-guided synergistic chemo-photothermal therapy (Figure 1). In this strategy, biodegradable and biocompatible MPDA with uniform size distribution of 150 nm have been successfully prepared through a nanoemulsion assembly approach and then served as a carrier for efficient encapsulation of hydrophobic perfluoropentane (PFP). Then, the amino-containing DOX can be immobilized stably on the surface of MPDA by Schiff base reaction to prevent the leaking of PFP during the blood circulation. By taking advantage of PDA, the obtained PFP@MPDA-DOX nanoplatform exhibits remarkable NIR absorption and high photothermal conversion efficiency (*η* = 45.6%), thus possess ideal ability for PA imaging as well as effective PTT. Upon NIR laser irradiation of PFP@MPDA-DOX, the mild hyperthermia will induce the liquid-gas phase transition of PFP, and leads to the generation of bubbles, which can not only promote the tumor cell uptake of nanotheranostics but also can be used as a contrast agents for ultrasound (US) imaging. *In vitro* and *in vivo* evaluations demonstrated the potential of the multicomponent nanoplatform for multimodel imaging-guided combinatorial cancer chemo-photothermal therapy. Prospectively, the developed MPDA-based nanoplatforms held great potential for cancer nanotheranostics, as well as further explore the application of polydopamine-based nanomaterials in nanomedicine.

## Materials and methods

### Materials

Ammonia aqueous solution (30%), acetone, and ethanol were purchased from the Sinopharm Chemical Reagent Co. (Shanghai, China). 1,3,5-trimethylbenzene (TMB), dopamine hydrochloride (DA), and perfluoropentane (PFP) were purchased from the Aladdin Reagent Co. Ltd. (Shanghai, China). Pluronic®F127, calcein AM, 3-(4,5-dimethylthiazol-2-yl)−2,5-diphenyltetrazolium bromide (MTT), doxorubicin hydrochloride (DOX⋅HCl) and propidium iodide (PI) were obtained from Sigma-Aldrich (St Louis, MO). Dulbecco’s modified Eagle medium (DMEM), penicillin-streptomycin solution, fetal bovine serum (FBS), 0.05% trypsin-EDTA, phosphate buffered saline (PBS), and 4′,6-diamidino-2-phenylindole (DAPI) were soured from Thermo Scientific (Beijing, China). All of cells were obtained from the Bogoo Biological Technology Co., Ltd (Shanghai, China). All chemicals were of analytical purity and used without additional purification.

### Synthesis of MPDA NPs

MPDA NPs were prepared according to the published methods by using Pluronic F127 as a template (Peng et al., 2019; Wu, Duan et al., 2019a). Briefly, 0.2 g of DA and 0.5 g of F127 were dissolved in 50 mL of mixed solution that containing deionized water and ethanol (1:1, v/v) under stirring. Then, TMB (1.0 mL) was added slowly and then stirred at room temperature to obtain a nanoemulsion system. After that, 2.5 mL of ammonia solution was introduced slowly with continuous stirring for 30 min, and the products were centrifuged (13,000 rpm), washed several times with ethanol and water. To remove the template F127, the product was re-dispersed in 50 mL of ethanol and acetone (2:1, v/v) and sonication for 1 h. This process was repeated three times to obtain the MPDA NPs.

### Synthesis of DOX-gated MPDA (MPDA-DOX) NPs

The amino-containing DOX can be immobilized stably on the surface of MPDA by Schiff base reaction (Li et al., 2019b). For the preparation of MPDA-DOX, the obtained MPDA NPs (50 mg) was mixed with DOX (10 mg) in 50 mL of tris buffer (10 × 10^−3 ^M, pH 8.5) with stirring for 2 h. Then, the final products were purified by centrifugation (10,000 rpm) to remove excess precursors, and the precipitates were re-dispersed in 40 mL of water for further use.

### Preparation of PFP@MPDA-DOX

For the synthesis of PFP@MPDA-DOX, 50 mg of MPDA-DOX NPs was added in an Eppendorf tube and the air in the systems was eliminated by vacuum pump. Afterwards, 150 μL of PFP was injected into the tube and sonication for 2 min in an ice bath to form PFP@MPDA-DOX. Then, the product was re-dispersed in PBS (10 mL) for further use.

### Characterization

The morphologies of MPDA NPs were observed by transmission electron microscopy (TEM, JEM-2100F, JEOL, Japan). N_2_ adsorption/desorption isotherms and pore size distribution were obtained by an ASAP 2020 absorption analyzer (Micromeritics Instruments Corporation, Atlanta, GA). The zeta potential and hydrodynamic diameters were measured on Zetasizer Nano ZS90 (Malvern Instruments Ltd., Malvern, UK). The ultraviolet-visible-near-infrared (UV-Vis-NIR) absorption spectra were recorded on UV-1700 Spectrophotometer (Shimadzu, Japan). The chemical compositions of the NPs were analyzed by Nexus 470 Fourier transform infrared spectrophotometry (FTIR, Nicolet, Madison, WI). The laser was acquired on an 808 nm NIR laser (Shanghai Xilong Optoelectronics Technology Co. Ltd., Shanghai, China). The thermal images and temperature changes were recorded by an infrared thermal imaging camera (FLIR E50, Fluke, Everett, WA).

### Photothermal properties of the MPDA NPs

The photothermal properties of MPDA NPs were investigated by recording the temperature changes with various concentrations of NPs (0–1000 µg mL^−1^) under an 808 nm NIR laser irradiation with laser power density of 1.0 W cm^−2^. Also, the MPDA NPs (600 µg mL^−1^) was exposed to the 808 nm laser at different power densities (0.25–1.25 W cm^−2^). Meanwhile, the thermal stability of MPDA NPs was evaluated by exposing to an 808 nm laser at a power density of 0.75 W cm^−2^ for three on-off cycles. Also, the photothermal conversion efficiency (*η*) of MPDA NPs was calculated by the previous methods (Ji et al., 2018; Zhen et al., 2018).

### pH and NIR-triggered DOX release from PFP@MPDA-DOX

To determine the DOX release behavior, 1 mL of PFP@MPDA-DOX dispersion (2 mg mL^−1^) was introduced to a dialysis bag (MWCO = 10 kDa), then shaken in 9 mL PBS at different pH (pH = 7.4 or 5.0). At the indicated time interval, 500 μL of the outside release media was taken out and same volume of fresh buffers was then added to the system. The released amount of DOX was examined with an UV-Vis spectrometer at 490 nm. To study NIR-triggered release of DOX, the experiments were performed at pH =7.4 and 5.0 and then treated with an 808 nm NIR laser irradiation (5 min, 1 W cm^−2^).

### Measurement of bubble release from PFP@MPDA-DOX

0.5 mL PFP@MPDA-DOX PBS solution (6 mg mL^−1^, pH = 5.0) was stored in a glass vial. The samples were covered with a coverslip and then treated with NIR laser irradiation (1.0 W cm^−2^) for 3 min. After that, the region of exposure was imaged under an optical microscope.

### *In vitro* PA/US imaging

*In vitro* PA and B-mode US imaging were realized by Vevo 2100 LAZR system (VisualSonics Inc., New York, NY). For *in vitro* PA imaging, PFP@MPDA-DOX NPs dispersions at different concentrations (0.5, 1, 2, 5 and 10 mg mL^−1^) were injected into Eppendorf tubes and the images were obtained by PA imaging system. Meanwhile, PFP@MPDA-DOX NPs was dispersed in PBS at concentrations of 2 and 10 mg mL^−1^. The US images and gray value of PFP@MPDA-DOX NPs were recorded on a PA/US imaging system with a mechanical index at 0.07 and a frequency at 40 MHz. The NPs for US imaging were observed before and after irradiation.

### Cell culture and bubble-enhanced cellular uptake

HUVEC, PC3, HepG2, and L929 cells were incubated in RPMI-1640 (HUVEC and PC3) or DMEM (HepG2 and L929) medium supplemented with containing 10% FBS and 1% antibiotics (penicillin-streptomycin), and cultured in a 5% CO_2_ atmosphere at 37 °C.

Intracellular uptake assay was performed by using confocal laser scanning microscopy (CLSM) and flow cytometry analysis. PC3 cells were seeded into 6-well plates at 2 × 10^5^ cells per well and incubated for 24 h. The old culture medium was replaced with fresh medium containing free DOX or PFP@MPDA-DOX NPs at the DOX concentration of 5 μg mL^−1^, and incubated for another 6 h. After 2 h incubation, part of cells was irradiated with an 808 nm laser (1.0 W cm^−2^) for 5 min. After the incubation was continued for totally 6 h, the cells were washed with PBS, fixed with 4% paraformaldehyde for 0.5 h and then stained with DAPI. The fluorescent images were observed by using CLSM (Olympus Fluoview FV-1000, Tokyo, Japan). For flow cytometry, the cells were seeded in glass-bottom culture dishes at a density of 5 × 10^4^ cells/dish and incubation for 24 h. The cells were treated with above-mentioned method. After that, the cells were digested with trypsin, harvested, and washed with PBS. Furthermore, the intracellular fluorescence of DOX was analyzed by using a flow cytometer (FACScan, Becton Dickinson, CA).

### *In vitro* combined antitumor efficiency of PFP@MPDA-DOX

The biocompatibility of MPDA NPs was evaluated using different cancer cells, including HUVEC, PC3, HepG2, and L929 cells. Briefly, these four different cell lines (5 × 10^3^ cells/well) were seeded in 96-well plates for 24 h. Subsequently, the medium was replaced with fresh medium containing different concentrations of MPDA NPs for another 24 h. The cells treated with DMEM medium were used as control. Finally, cell viability was evaluated by standard MTT assay according to the manufacturer’s instructions.

To study chemo-photothermal therapeutic efficacy, PC3 cells at a density of 5 × 10^3^ cells/well were seeded into 96-well plates and then treated as following: free DOX, MPDA, MPDA-DOX and PFP@MPDA-DOX at different concentrations of MPDA and same concentrations of DOX of 5 μg mL^−1^. The cells were further incubated for another 4 h. After that, the cells were treated with 808 nm NIR irradiation (1 W cm^−2^, 5 min) and after incubation for an additional 24 h. The cell viabilities were detected by MTT assay. Meanwhile, the synergistic effect of chemo-photothermal was evaluated by combination index (CI) analysis and the CI value was calculated according to the previous study (Xu, Teng, et al., 2018).

To further verify the MTT results, the calcein-AM/PI assay was performed. Briefly, PC3 cells (5 × 10^3^ cells/well) were seeded into 96-well plates and incubated for 24 h, followed by treated with the same steps as above. Then, the cells after different treatments were stained with calcein-AM (20 μM) and PI (4 µM) solution in PBS buffer solution for 20 min. Finally, the cells were washed three times with PBS and imaged by using a digital microscope.

### Animal tumor model and *in vivo* multimodal imaging

BALB/c nude mice (15–20 g, female) were obtained from Shanghai Slac Laboratory Animal Center (Shanghai, China). All animal experiments were approved by the Institutional Animal Ethical and Welfare Committee of Kunming Medical University (Kunming, China). To establish the xenograft tumor models, 200 μL of PBS containing PC3 cells (2 × 10^6^) were subcutaneously injected into right flank area of the mice. The tumor size was monitored every other day by a caliper and the tumor volume (*V*) was calculated as follows: *V* = 0.5 × *L* × *W*^2^ (*L*, *W* are the represented length and width of the tumor, respectively). The mice were used for further *in vivo* experiments when the tumor volume had reached approximately 100 mm^3^.

For US imaging *in vivo*, 100 μL PFP@MPDA-DOX (6 mg mL^−1^) was intravenously injected nto the tumor-bearing mice via tail vein. The tumors region was irradiated with a 808 nm NIR laser (1 W cm^−2^) for 5 min after 24 h injection. Then, US imaging measurements were conducted before and after NIR laser irradiation. Additionally, PA imaging was performed at different time points (0, 4, 12, and 24 h) after injection. All PA and US images were analyzed and collected with a Vevo LAZR imaging system (VisualSonics Inc. New York, NY) with a mechanical index at 0.07 and a frequency at 40 MHz.

### *In vivo* antitumor efficiency and biosafety

For *in vivo* combined therapy, when the tumor size reached ∼100 mm^3^ (day 0), PC3 tumor-bearing mice were randomly divided into five groups (6 animals per group): (1) PBS; (2) free DOX; (3) MPDA + Laser; (4) MPDA-DOX + Laser; (5) PFP@MPDA-DOX + Laser. The injection was conducted every 3 days with a dosage of 10 mg kg^−1^. For groups 3, 4, and 5, the tumors were exposed to 808 nm laser irradiation (1.0 W cm^−2^, 10 min) at 12 h post-injection. The thermal images of mice were recorded and monitored by an infrared thermal imaging camera (FLIR E50, Fluke, Everett, WA) during the treatment. Furthermore, the body weights of mice were recorded every 2 days. At the end of various treatments, the tumors were collected to make paraffin section for hematoxylin and eosin (H&E) and terminal deoxynucleotidyl transferase dUTP nick end labeling (TUNEL) staining following to the manufacturer’s protocol. Also, for the *in vivo* biosafety study, the H&E staining of the organs (heart, liver, spleen, lung, and kidney) was conducted at 14 days post-injection.

### Statistical analysis

All the experiments were carried out at least three times. The experimental data are expressed as mean ± standard deviation (SD). Statistical analysis was analyzed with the SPSS software using one-way analysis of variance (ANOVA) followed by post hoc Tukey’s test. Statistical values are indicated in figures according to the following scales: *p < .05, **p < .01, and ***p < .001.

## Results and discussion

### Preparation and characterization of PFP@MPDA-DOX NPs

**Figure 2.**
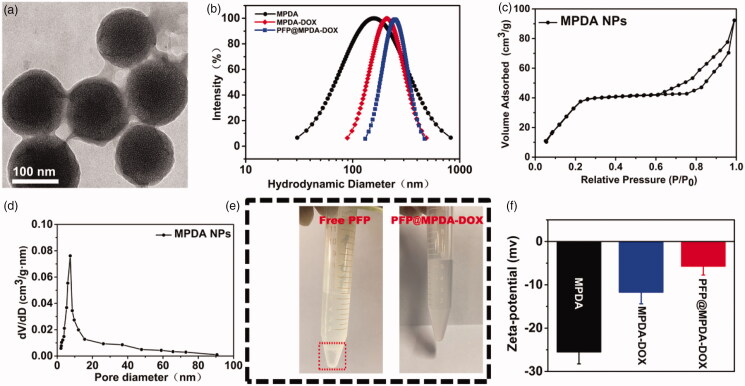
(a) TEM images of MPDA NPs. (b) Particle-size distribution of MPDA, MPDA-DOX, and PFP@MPDA-DOX. (c) N_2_ adsorption-desorption isotherms. (d) Pore size distribution of MPDA NPs. (e) Photographs of free PFP and PFP@MPDA-DOX in PBS. (f) Zeta potential of MPDA, MPDA-DOX, and PFP@MPDA-DOX NPs (*n* = 3).

The synthetic procedure of the PFP@MPDA-DOX NPs is presented in Figure 1. MPDA NPs were prepared through a versatile nanoemulsion assembly approach according to the previous study (Peng et al., 2019; Wu, Duan, et al., 2019). The Pluronic F127 and TMB were served as the organic templates. Then, the assembly process was performed by using TMB to regulate the interfacial interaction (π–π stacking) between dopamine and F127. The templates were removed and the MPDA NPs with uniform size distribution of around 124 nm was obtained (Figure 2(a)). The MPDA NPs also possessed the well-defined spherical morphology and mesoporous structure, which were clearly observed from TEM images. MPDA NPs exhibited a uniform hydrodynamic size of 158 ± 4 nm in PBS (Figure 2(b)). The mesoporous characteristic of the MPDA NPs was measured through N_2_ absorption-desorption isotherms. As shown in Figure 2(c,d), the resultant MPDA NPs show the surface area of 51.3 m^2^ g^−1^ and pore size of 7.44 nm, which was significantly larger than that of PDA NPs without porous structure (0.213 m^2^ g^−1^) (Wu et al., 2019). This endows the MPDA NPs promising for sufficient encapsulation of hydrophobic payloads.

Next, antitumor drug DOX was served as the ‘gatekeeper’ to introduce into the MPDA NPs. Subsequently, the amino-containing DOX can be immobilized stably on the surface of MPDA by Schiff base reaction. After modification with DOX molecule, the hydrodynamic diameter of NPs slightly increased to ≈171 nm (Figure 2(b)). The DOX-gated MPDA were tested by FT-IR analysis, as depicted in Figure S1 in Supplementary Material, the characteristic peaks at 1740 cm^−1^ were observed in spectra of MPDA-DOX, which was assigned to C=O stretching vibration of DOX, demonstrated the success of DOX modification.

**Figure 3.**
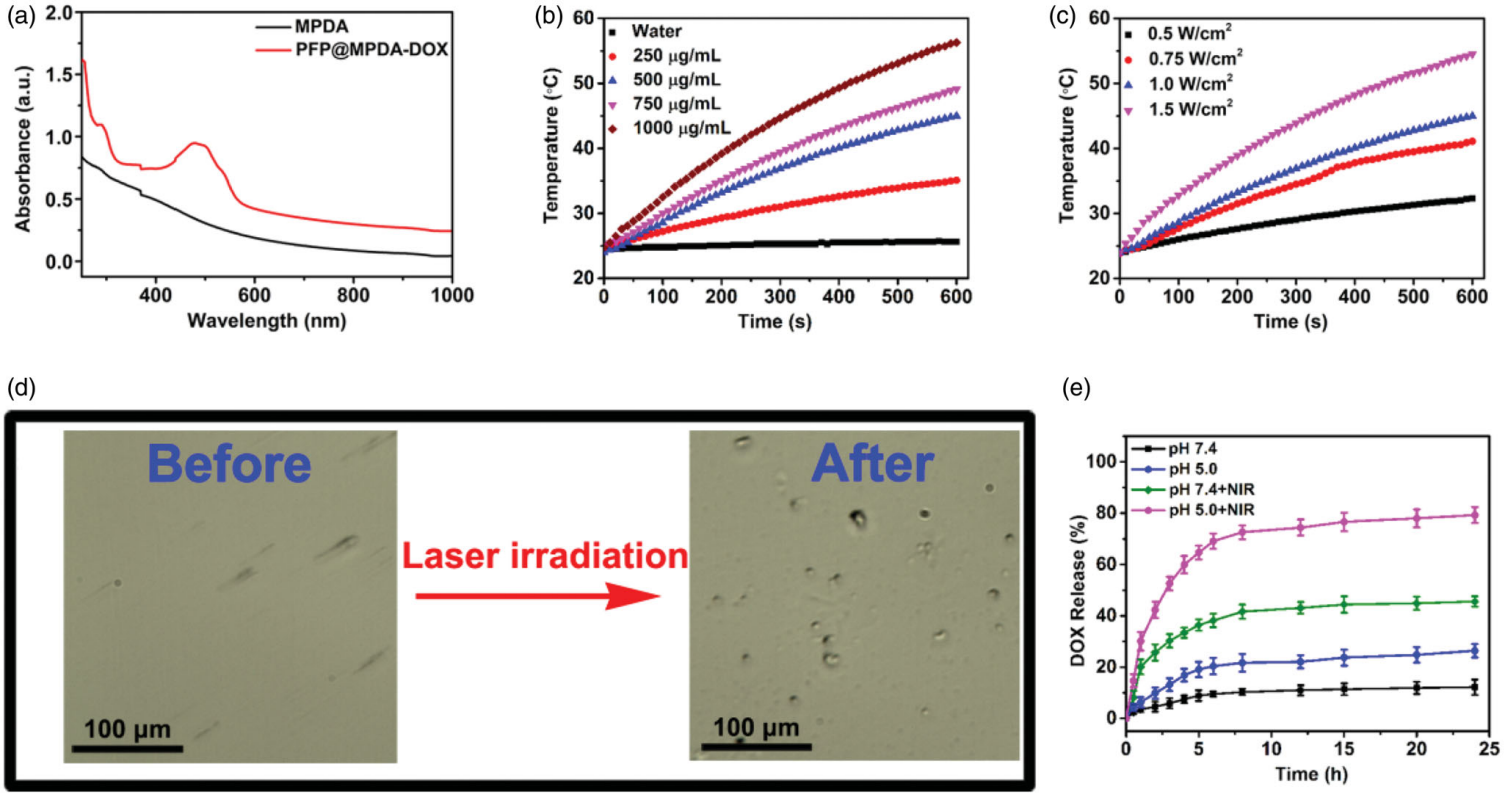
(a) UV-Vis spectra of MPDA and PFP@MPDA-DOX NPs. (b) Temperature increase profiles of MPDA aqueous solution at various concentrations upon NIR laser irradiation (power density: 1.0 W cm^−2^) for 10 min. (c) Temperature increase profiles of MPDA aqueous solution upon NIR laser irradiation with different power densities (1000 μg mL^−1^) for 10 min. (d) Optical microscopy images of PFP@MPDA-DOX dispersion before and after NIR laser irradiation for 3 min. (e) DOX release from PFP@MPDA-DOX in PBS at pH 5.0 and 7.4 with or without NIR laser irradiation (1.0 W cm^−2^).

Then, MPDA-DOX NPs is served as nanocarrier for encapsulating of hydrophobic cargos (PFP) via vacuum impregnation (Jia et al., 2015; Lu et al., 2018). Obviously, a clear phase separation phenomenon was observed when the introduction of free PFP into PBS (Figure 2(e)). By contrast, the resulted PFP-loaded MPDA-DOX NPs exhibited good dispersity in PBS, confirming that PFP could be encapsulated into the MPDA. Based on the DLS data, the hydrodynamic size was increased along with the synthetic steps (Figure 2(b)). Moreover, the zeta potentials of MPDA and PFP@MPDA-DOX NPs were determined to be −25.6 and −6.4 mV, respectively (Figure 2(f)). Furthermore, UV-Vis spectra of PFP@MPDA-DOX exhibited a characteristic absorption peak of DOX at ≈480 nm, suggesting the successful gating of DOX (Figure 3(a)). Also, the DOX content in the PFP@MPDA-DOX NPs was calculated to be 26.1 wt%. All these results confirm the successful fabrication of the PFP@MPDA-DOX NPs.

### NIR-triggered PFP bubble release and pH/NIR-responsive DOX release of PFP@MPDA-DOX NPs

Based on the UV-Vis spectra, MPDA NPs exhibits a strong NIR absorption, which makes it suitable for efficient photothermal conversion. Thus, the photothermal performance of MPDA NPs was evaluated. The temperature of MPDA dispersion increased in both concentration and power-dependent manner (Figure 3(b,c)). The temperature of MPDA dispersion (1000 µg mL^−1^) could be increased from 24.8 to 56.3 °C after irradiated to 808 nm laser (1.0 W cm^−2^) for 10 min, whereas the temperature of PBS was slightly increased to 25.6 °C under the same conditions. Notably, the *η* of MPDA NPs was also measured to be of 45.6%, which was obviously higher than most previously reported nanomaterial (Wang et al., 2016; Yang et al., 2016; Xing et al., 2017; Yang et al., 2018). Meanwhile, the photostability of MPDA were further assessed and negligible change of the temperature of the aqueous solution of MPDA (500 µg mL^−1^) was observed during three cycles of irradiation (Figure S2 in Supplementary Material). All these results certified that MPDA NPs could be a promising agent for NIR laser induced hyperthermia.

The high photothermal conversion performance of MPDA NPs also makes the temperature-responsive liquid-gas phase transformation of PFP. The NIR-triggered PFP bubble release was measured in PBS at pH 7.4. As shown in optical microscopy images (Figure 3(d)), the amount of microbubbles rapidly increased along with the increase of the laser irradiation time, while no bubbles were found in the aqueous solution of MPDA-DOX with laser irradiation. This verified that the loading of PFP liquid could be gasified by induced hyperthermia by NIR laser irradiation of MPDA, and the induced microbubbles could be utilized as contrast for US imaging (Tang et al., 2017).

The excellent photothermal properties also make the DOX gatekeeper can be released in response to hyperthermia. The release profiles of DOX from PFP@MPDA-DOX NPs were measured in PBS (pH 5.0 and 7.4) in the presence or absence of NIR laser irradiation (1 W cm^−2^, 5 min). As shown in Figure 3(e), less than 13% of DOX is released from the PFP@MPDA-DOX at pH 7.4 over 20 h, indicating the obtained NPs is stable in normal physiological environment. In contrast, the rate the cumulative release of DOX dramatically increased to 26.4% within 24 h at pH 5.0. The pH-responsive release behavior can be attributed to the biodegradation of PDA framework under the acid condition, which is benefit for effective cancer chemotherapy. In addition, the NIR laser irradiation triggered release performance was further investigated. As expect, the DOX release rate can reach to 45.6% at pH 7.4 and 79.3% at pH 5.0 during 24 h under laser irradiation, indicating a NIR-responsive drug release property. These results indicated that the DOX as a gatekeeper could not only improve the PFP delivery efficiency but also can be controlled accurately by dual stimuli.

### *In vitro* PA/US imaging

**Figure 4.**
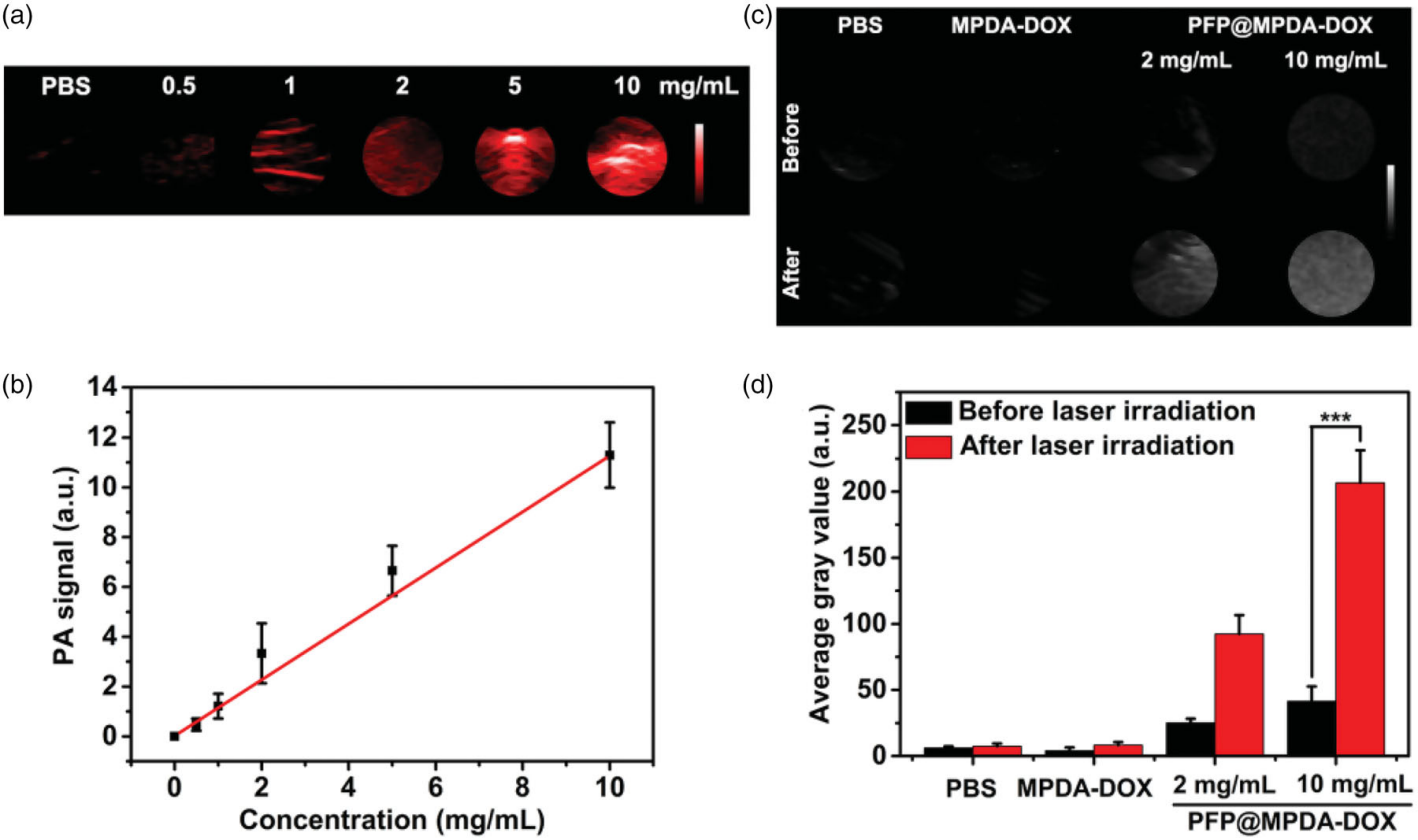
(a) PA images and (b) PA values of PBS and PFP@MPDA-DOX NPs at different concentrations. (c) US images in B mode and (d) the corresponding average gray values of water, MPDA-DOX and PFP@MPDA-DOX NPs before and after NIR irradiation.

As an emerging imaging technique, PA imaging has recently attracted much interest in nanomedicine owing to its non-ionization, high spatial resolution, as well as the deep tissue penetration (Chen, Wang, et al., 2014; Kim et al., 2018). The strong NIR absorption and effective photothermal effect also makes PFP@MPDA-DOX NPs suitable for PA imaging. We further investigated PA imaging performance of PFP@MPDA-DOX NPs at different concentrations. As presented in Figure 4(a), a concentration-dependent enhancement of PA signal was observed. Clearly, quantitative analysis result shows a good linear correlation between the PA signal and the concentration of the aqueous solutions (Figure 4(b)), endowing it is suitable for PA imaging. The NIR laser-responsive microbubbles generation has been confirmed above, the US imaging properties of PFP@MPDA-DOX NPs with or without laser irradiation (808 nm, 1.0 W cm^−2^) were investigated. From B-mode US images (Figure 4(c)), no obvious changes of US signals were observed for PBS and MPDA-DOX with laser irradiation. In contrast, the echo signals of bubbles in PFP@MPDA-DOX group were significantly enhanced after laser exposure, which may be attributed to the liquid-gas phase transformation of PFP by hyperthermia. Correspondingly, the average gray value of PFP@MPDA-DOX after NIR irradiation was almost quintuple higher than that before laser irradiation (Figure 4(d)). This confirmed that our PFP@MPDA-DOX nanotheranostics has good potential for US/PA dual-modality imaging.

### *In vitro* cellular uptake

**Figure 5.**
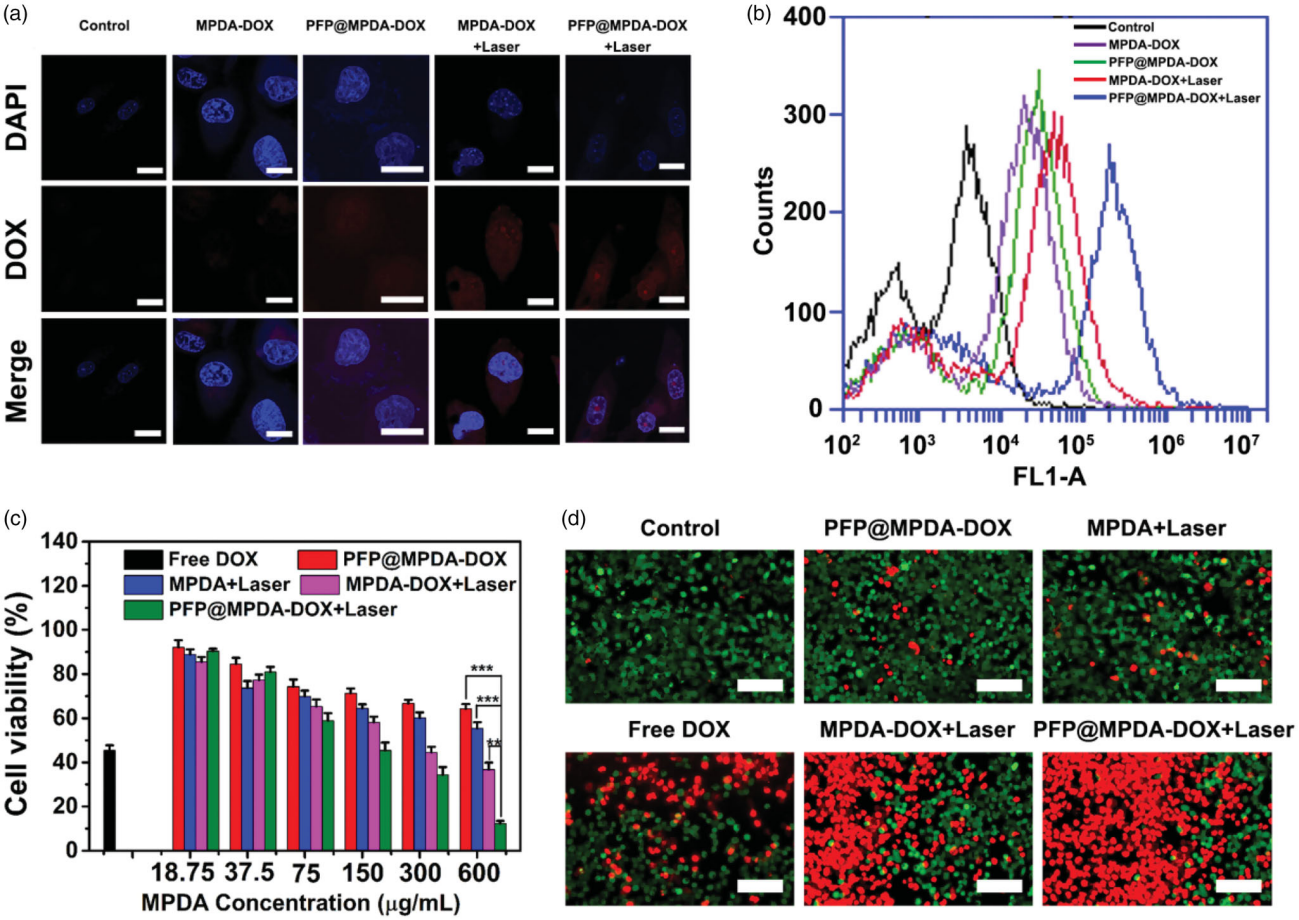
(a) CLSM images of PC3 cells after incubated with MPDA-DOX and PFP@MPDA-DOX without or with NIR laser irradiation. Scale bar: 25 μm. (b) Flow-cytometry analyses of MDA-MB-231 cells incubated with MPDA-DOX and PFP@MPDA-DOX without or with NIR laser irradiation. (c) Cell viabilities of PC3 cells after different treatments, including free DOX, PFP@MPDA-DOX only, MPDA + Laser, MPDA + Laser, PFP@MPDA-DOX + Laser at varied MPDA concentrations. Data are given as the mean ± SD (*n* = 5), **p < .01 and ***p < .001. (d) Fluorescence microscopic images of PC3 cells stained by calcein AM (green) and PI (red) after different treatments. Scale bars: 100 µm.

The bubbles enhanced cellular uptake of NPs have been proved in many previous reports due to the emergence of permeable defects in lipid bilayers by the intracellular generated microbubbles (Chen, Chuang, et al., 2014; Wu, Williams, et al., 2019). Therefore, the cellular uptake efficiency of the presented PFP@MPDA-DOX was monitored by CLSM and flow cytometry, respectively. Obviously, a relatively weaker DOX fluorescence in PC3 cells that incubated with MPDA-DOX or PFP@MPDA-DOX (Figure 5(a)). In contrast, MPDA-DOX combined with NIR laser irradiation could enhanced uptake to some extent, mainly due to the hyperthermia induced minor disruptions to cell membrane for improving uptake of NPs (Sun et al., 2013). Also, the fluorescent signal of DOX was further dramatically increased in the cells treated with PFP@MPDA-DOX with laser irradiation. This could be attributed to the generated PFP bubbles induced by the hyperthermia promoting the cellular uptake of NPs. Subsequently, quantitative analysis by flow cytometry further confirm the intracellular DOX fluorescence in PFP@MPDA-DOX was 1.96-fold higher than that in MPDA-DOX with laser irradiation at the same power density (Figures 5(b) and Figure S3 in Supplementary Material). These results clearly verified the induced hyperthermia and bubble by PFP@MPDA-DOX could strongly enhance the cell uptake of NPs.

### *In vitro* synergistic therapy of PFP@MPDA-DOX

Encouraged by the above-mentioned results, *in vitro* anticancer effect was further evaluated. First, biocompatibility of MPDA NPs is the prerequisite for their therapeutic applications. Hence, the cytotoxicity of MPDA NPs was evaluated in various cells (HUVEC, PC3, HepG2, and L929) using the MTT assay. As expected, MPDA exhibited negligible toxicity on the four different cell lines, even at a high incubation concentration of 600 µg mL^−1^ (Figure S4 in Supplementary Material), indicated the good biocompatibility of MPDA NPs. After PFP loading and DOX gating, the *in vitro* therapeutic efficacy of chemotherapy and PTT against PC3 cells was then evaluated. As shown in Figure 5(c), a MPDA concentration-dependent cell inhibition toward PC3 cells were observed for all treatments that treated with NIR irradiation (808 nm, 1.0 W cm^−2^) for 5 min. More than 60% of PC3 cells were killed by MPDA-DOX NPs combined with laser irradiation, indicating the high therapeutic outcomes of synergistic chemo-photothermal therapy. More importantly, the cell viability of PFP@MPDA-DOX with NIR irradiation was further decreased to 12.3% at MPDA concentration of 600 μg mL^−1^, which is much lower than MPDA-DOX under laser irradiation group. The enhanced inhibition efficiency is mainly due to the synergistically enhanced cell uptake by the generation of PFP bubble. Meanwhile, the CI of PTT and PDT was calculated to be 0.29, verifying a potent synergistic effect. Therefore, PFP@MPDA-DOX NPs can be used as a biocompatible nanoplatform for highly efficient chemotherapy and PTT. These results can also be verified by the calcein AM/PI stained assay (Figure 5(d)). It was found that cells treated with PFP@MPDA-DOX NPs combined with NIR laser exhibits an intense of red fluorescence, which is more notable than other treatments groups.

### *In vivo* US/PA imaging

**Figure 6.**
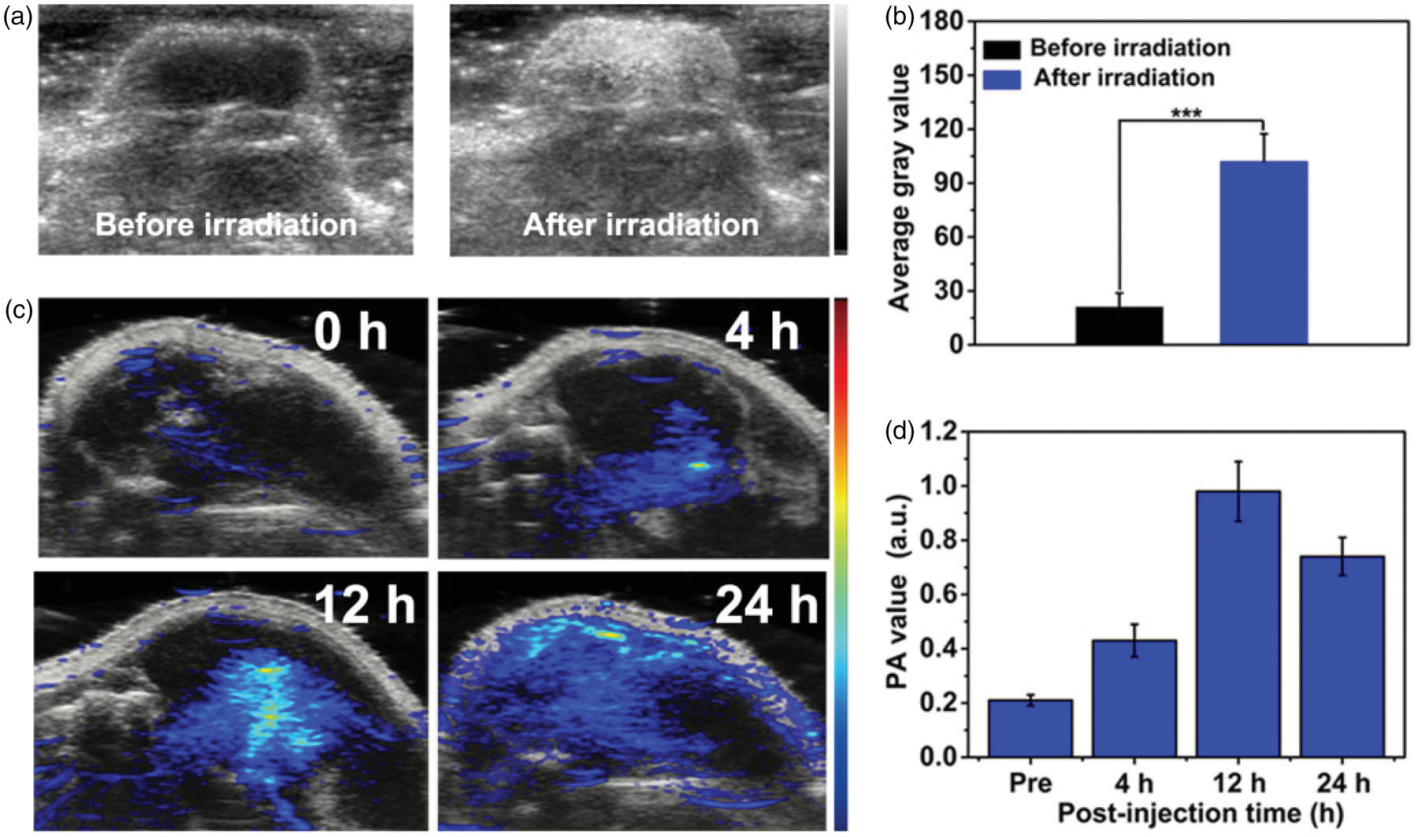
(a) *In vivo* B-mode US images and (b) the corresponding gray values of PC3 tumor injected with PFP@MPDA-DOX before and after NIR laser irradiation 24 h post-injection. (c) PA images and (d) the mean PA signal intensity of PC3 tumor-bearing mice pre- and post-intravenous injection of PFP@MPDA-DOX NPs at different time points (4, 12, and 24 h).

Encouraged by the good US/PA imaging performance of PFP@MPDA-DOX *in vitro*, the feasibility of PFP@MPDA-DOX for *in vivo* imaging was performed on PC3 tumor model after *i.v.* injection with PFP@MPDA-DOX (6 mg mL^−1^). For PA imaging *in vivo*, the PC3 tumor-bearing mice were intravenously injected with PFP@MPDA-DOX and the US signal was recorded before and after NIR laser irradiation (Figure 6(a)). Only faint US signal in the tumor can be observed before laser irradiation. This is attributed to the fact that the physiological temperature (37 °C) is not high enough to cause the liquid-gas transformation of PFP *in vivo* due to the blood/intratumor pressure (Li et al., 2018a; Lu et al., 2018). Excitingly, the US signal in B mode was significantly enhanced after NIR laser irradiation. The reason for this could be the generation of PFP nanobubbles due to the regional temperature was turned to about 50 °C in tumor, leading to the continuous coalescence into microbubbles. Meanwhile, the average gray value of tumor sites after laser irradiation was much stronger than the pre-injection value (Figure 6(b)). Furthermore, the potential of PFP@MPDA-DOX NPs as a PA imaging contrast agent was investigated after intravenous injection of NPs. As depicted in Figure 6(c), the PA signal from tumors region showed a time-dependent enhancement manner, reached the highest intensity at 12 h post-injection and a strong PA signal of tumor was still observed at 24 h. Subsequently, the quantitative PA signal was analyzed, and the results were consistent with the PA images (Figure 6(d)). These results illustrated that our designed PFP@MPDA-DOX could perform a high tumor accumulation and simultaneously acted as an excellent contrast agent for US/PA imaging.

### *In vivo* antitumor efficacy

**Figure 7.**
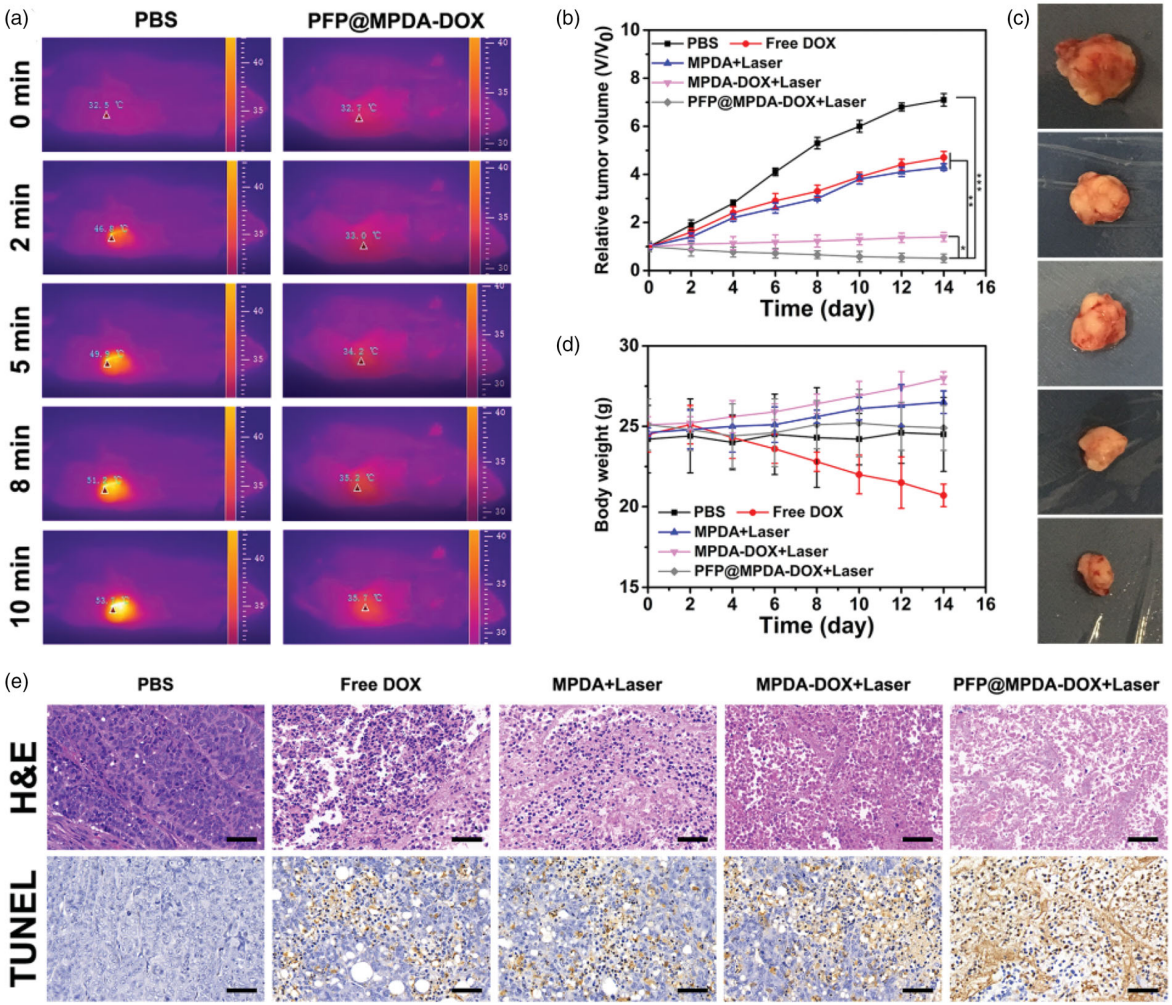
(a) *In vivo* thermal imaging of mice after treated with PBS and PFP@MPDA-DOX at 12 h. (b) Relative tumor volume curves of PC3 tumor bearing nude mice during different treatments. (c) Digital photographs of the representative excised tumors mice after various treatments. (d) Body weight of PC3 tumor bearing nude mice after different treatments. (e) H&E and TUNEL staining of tumor excised from mice in different groups. Scale bars: 50 µm.

Motivated by the above excellent results, we then evaluate *in vivo* antitumor study to demonstrate the synergistic therapy of PFP@MPDA-DOX on PC3 tumor-bearing mice model. The mice were randomly divided into five groups: (1) PBS; (2) free DOX; (3) MPDA + Laser; (4) MPDA-DOX + Laser; (5) PFP@MPDA-DOX + Laser. For the NIR groups, the mice were exposed to 808 nm laser irradiation (1.0 W cm^−2^, 10 min) at 12 h post-injection. Meanwhile, the *in vivo* thermal images were recorded using an IR thermal camera (Figure 7(a)). The temperature of tumor treated with PFP@MPDA-DOX + NIR can rise to 53.5 °C within the laser irradiation, while PBS-treated mice remained stable at physical temperature (Figure S5 in Supplementary Material).

During the treatments, the tumor volume and body weights were monitored. As shown in Figure 7(b), compared with saline treatment, the groups of free DOX showed slight inhibition tumor growth due to the chemotherapy effectiveness. Better therapeutic effects were observed in MPDA group (PTT), demonstrating the potential application of MPDA for PTT. Additionally, tumor-inhibiting effect in group (4) also exhibits superiority than that of group 1–3, revealing an *in vivo* synergistic effect of MPDA-DOX NPs when exposed to laser irradiation. Notably, the mice in PFP@MPDA-DOX + Laser showed the most efficient inhibition on tumor growth compared with other treatment groups, demonstrating a remarkable synergistic therapeutic effect owing to the PFP microbubble enhanced cellular uptake. Meanwhile, the digital photos of the excised tumors after different treatments (Figure 7(c)) visually indicated the excellent therapeutic outcomes of PFP@MPDA-DOX NPs. Simultaneously, the mice treated with free DOX showed an obvious drop in body weight, which may be due to the side effect of DOX. In contrast, negligible body weight changes were noted in other treatments (Figure 7(d)), indicating good tolerance of formulations in mice.

To further clarify the antitumor efficacy, tumor sections from the sacrificed mice after various treatments were stained with H&E and TUNEL. In H&E staining assays (Figure 7(e)), significant large-area apoptosis/necrosis regions can be observed in the treatment of PFP@MPDA-DOX + NIR, while no notably affected or only partially destroyed was displayed in other groups. Analogously, the maximum ratio of apoptotic cells was detected from the TUNEL stained images in synergistic group (Figure 7(f)), which is in accordance with H&E results, indicating the high efficacy of synergistic chemo-PTT treatment.

Moreover, the low side effects of the PFP@MPDA-DOX NPs were further investigated by histology analysis. From H&E-stained images (Figure S6 in Supplementary Material), no obvious organ damage was observed for major organ sections (heart, liver, spleen, lung, and kidney) from PFP@MPDA-DOX-treated group, indicating a satisfactory safety and excellent biocompatibility of PFP@MPDA-DOX NPs.

## Conclusions

In summary, we have successfully developed an intelligent ‘all-in-one’ platform for US/PA imaging guided synergistic chemo-photothermal therapy of prostate tumors, which has been achieved by integrating a hydrophobic PFP into MPDA NPs. Through gated with antitumor drug, the as-prepared nanotheranostics (PFP@MPDA-DOX) could reasonably avoid the use of exogenous capping agents and showed pH/NIR-responsive payloads release. Importantly, the strong NIR absorption of MPDA results in NIR-responsive hyperthermia generation for PTT and PA, as well as further induce the liquid-gas phase transformation of PFP to realize the enhanced US imaging, which is conducive to accurate diagnosis and treatment. Both *in vitro* and *in vivo* therapeutic evaluations have confirmed the superior synergistic chemotherapy and PTT efficacy of our PFP@MPDA-DOX. Also, the excellent biocompatibility and low observable toxicity facilitated its applications in therapeutic applications. It is thus expected that design of DOX-gated MPDA strategy provides an efficient strategy to explore the application of polydopamine-based nanomaterials, particularly for enhanced dual-modality US/PA imaging guided chemo-PTT of cancer.

## Supplementary Material

Supplemental Material
