# Supplementary material for: Fabrication of doxorubicin-gated mesoporous polydopamine nanoplatforms for multimode imaging-guided synergistic chemophotothermal therapy of tumors
Source: Drug Deliv. 2020 Feb 24;27(1):367–77. doi: 10.1080/10717544.2020.1730523 (PMC7054968; doi:10.1080/10717544.2020.1730523)
Supplement: Supplemental Material [file IDRD_A_1730523_SM8253.docx]

**Supporting Information**

**Fabrication of doxorubicin-gated mesoporous polydopamine nanoplatforms for multimode imaging-guided synergistic chemophotothermal therapy of tumors**

Min Yang,^1^ Ningnan Zhang,^1^ Tao Zhang,^2^ Xian Yin,^1^ Jie Shen^1,*^

^1^Department of Urology, The First People’s Hospital of Yunnan Province, Kunming University of Science and Technology, Kunming, 650032, P. R. China

^2^School of Chemical Science and Technology, Yunnan University, Kunming, 650091, P. R. China

*Corresponding authors. Email: Sjkmyf2435@163.com

**
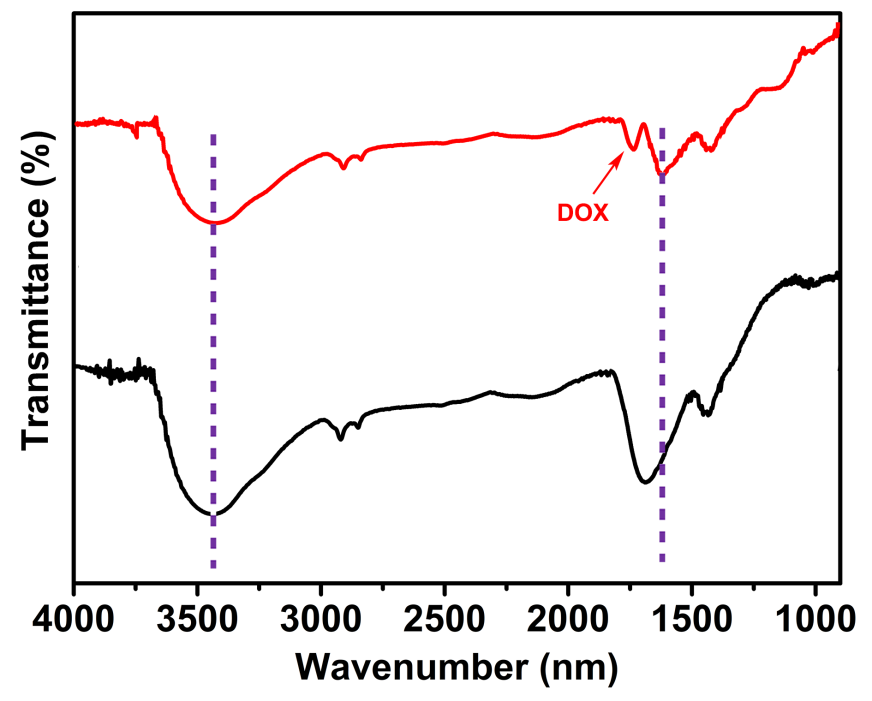
**

**Figure S1.** Fourier transform infrared (FT-IR) spectra of MPDA (black line) and MPDA-DOX NPs (red line).

**
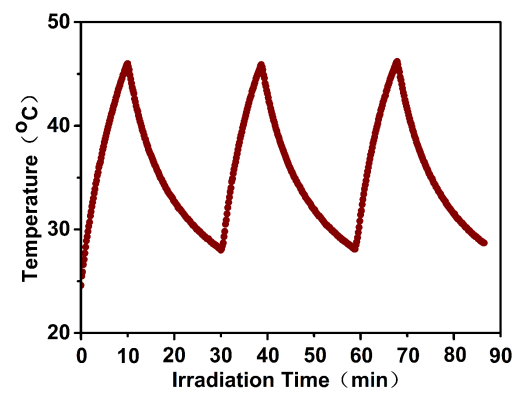
**

**Figure S2.** Photostability tests of MPDA NPs for three cycles of NIR laser irradiation.

**
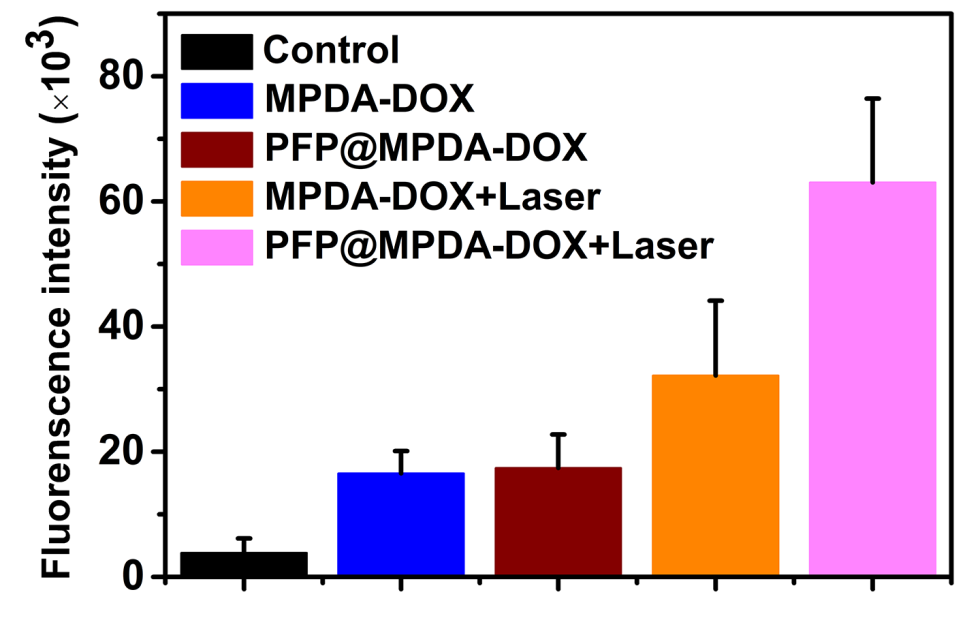
**

**Figure S3.** Corresponding relative mean fluorescence intensity of PC3 cells incubated with MPDA-DOX and PFP@MPDA-DOX without or with NIR laser irradiation.

**
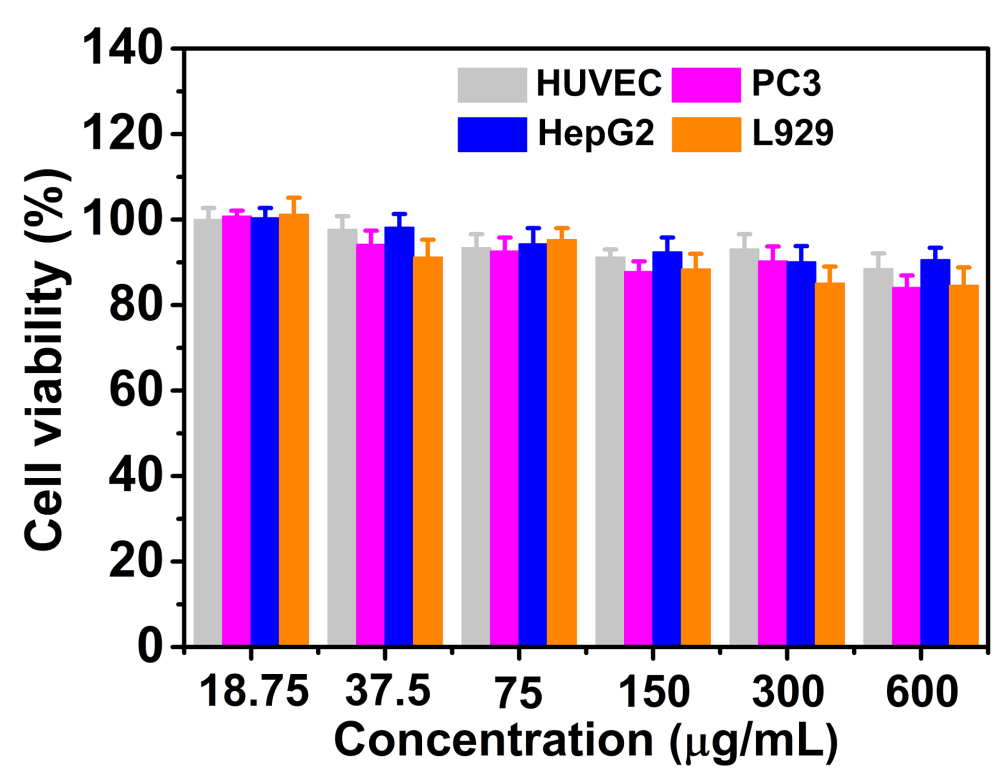
**

**Figure S4.** Cell viabilities of HUVEC, PC3, HepG2, and L929 cells after incubation with various doses of blank MPDA NPs for 24 h. Data are given as the mean ± SD (n = 5).

**
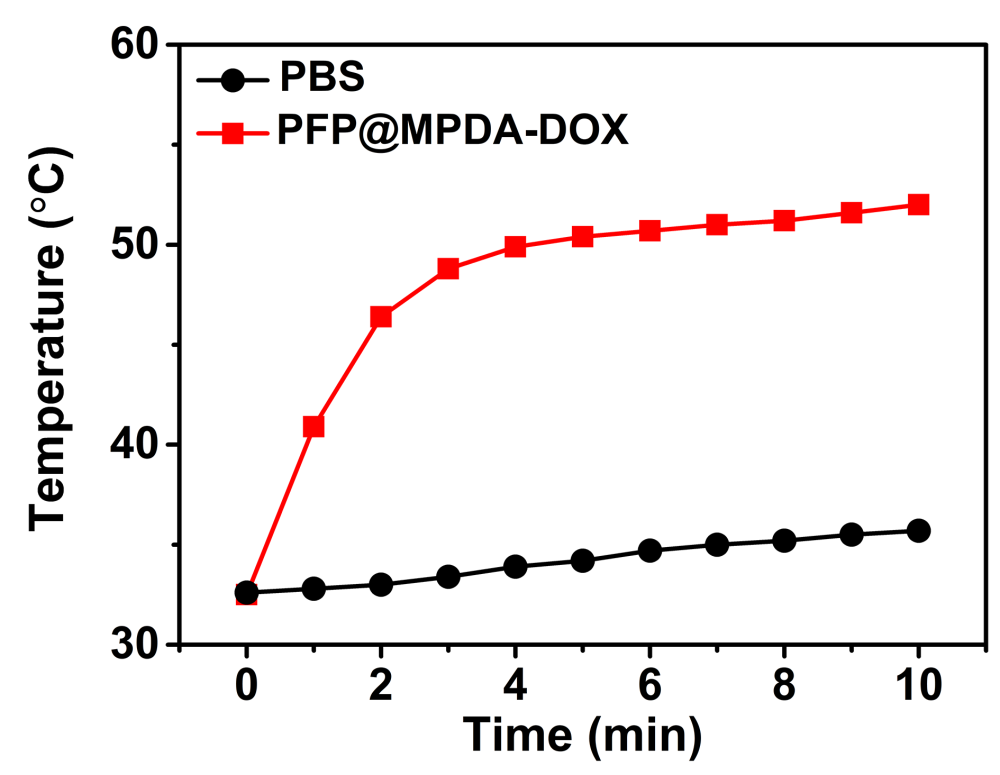
**

**Figure S5.** Temperature recording of tumor site upon 10 min 808 nm laser exposure (1.0 W cm^-2^) after intravenous injected with PBS and PFP@MPDA-DOX at 12 h.

**
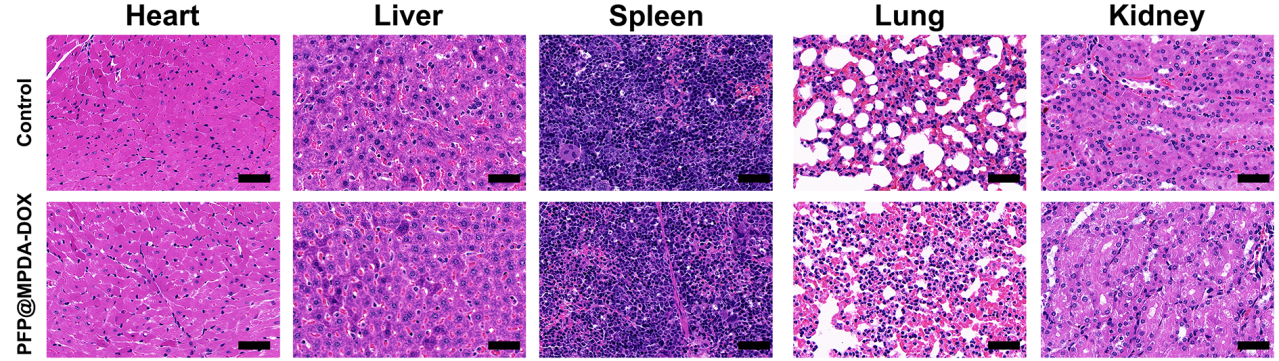
**

**Figure S6.** H&E stained histological images of major organs (heart, liver, spleen, lung, and kidney) from mice treated with PBA and PFP@MPDA-DOX NPs. Scale bar: 50 µm.
